# The role of alpha-actinin-4 in human kidney disease

**DOI:** 10.1186/s13578-015-0036-8

**Published:** 2015-08-18

**Authors:** Di Feng, Clark DuMontier, Martin R Pollak

**Affiliations:** Division of Nephrology, Department of Medicine, Beth Israel Deaconess Medical Center, Boston, MA 02215 USA; Department of Medicine, Boston University School of Medicine, Boston, MA 02118 USA

**Keywords:** Focal segmental glomerulosclerosis, Alpha-actinin-4, Kidney, Podocytes, Cytoskeleton protein

## Abstract

Mutations in the Alpha-actinin-4 gene (ACTN4) cause a rare form of familial focal segmental glomerulosclerosis in humans. Individuals with kidney disease-associated ACTN4 mutations tend to have mild to moderate proteinuria, with many developing decreased kidney function progressing to end stage kidney disease. All of the disease-causing ACTN4 mutations identified to date are located within the actin-binding domain of the encoded protein, increasing its binding affinity to F-actin and leading to abnormal actin rich cellular aggregates. The identification of ACTN4 mutations as a cause of human kidney disease demonstrates a key cellular pathway by which alterations in cytoskeletal behavior can mediate kidney disease. Here we review the studies relevant to ACTN4 and its role in mediating kidney disease.

## Background

Focal segmental glomerulosclerosis (FSGS) is a common histologic pattern of kidney injury, often associated with progressive chronic kidney disease and overt kidney failure. FSGS is defined histologically by sclerosis occurring in a portion of the glomerulus and affecting a subset of glomeruli. While these biopsy features define a pattern of injury, they do not define the underlying etiology [1]. Electron microscopy typically shows fusion of epithelial cell foot processes, or so-called foot process effacement. Common features of FSGS patients include proteinuria, edema, hypertension and hypercholesterolemia.

FSGS is found in about 20–25% of all renal biopsies [2, 3]. However, the incidence and prevalence of FSGS in children may be underestimated [4]. This is because many children with nephrotic syndrome (NS), defined as heavy proteinuria, hypoalbuminemia, and peripheral edema, are generally assumed to have minimal change syndrome and not subjected to renal biopsy. Even when a biopsy is performed, the characteristic lesion necessary for diagnosis might not be captured in the sample. It is estimated that the incidence of NS in children is between 2 and 4 new cases per 100,000 children per year, with biopsy-confirmed FSGS comprising 15–20% [4, 5]. The prevalence of FSGS continues to increase for unknown reasons [2, 5, 6]. FSGS is seen in approximately 2–5% of cases of end stage renal disease (ESRD) in adults and about 10% in children [7].

A number of etiologies have been associated with FSGS and are often used as the basis for further classification. Broadly, FSGS is divided into primary (idiopathic) and secondary forms. Secondary FSGS is thought to result from a wide range of systemic conditions, including reflex nephropathy, heroin use, decreased renal mass, hypertension, diabetes, obesity, and HIV infection [8, 9]. In comparison with secondary FSGS, primary FSGS is more likely to present with nephrotic-range proteinuria and have a poorer prognosis, with 50% progressing to ESRD over 3–8 years ([9, 10]. For FSGS patients with nephrotic-range proteinuria, the use of corticosteroids is associated with an increased likelihood of a remission [9, 11]. However, steroid resistance can be present in up to 50% of patients, and a prolonged steroid treatment is associated with significant side effects [9]. Therefore, there is great interest in understanding the molecular mechanisms underlying different forms of FSGS and kidney injury. Mutations in several genes are associated with familial forms of FSGS or nephrotic syndrome, including α-actinin-4 (here we use ACTN4 to designate the human gene or protein and Actn4 to designate the mouse gene or protein) [12], inverted formin 2 (INF2) [13], canonical transient receptor potential 6 (TRPC6) [14, 15], nephrin (NPHS1) [16], and podocin (NPHS2) [17]. This review will focus on ACTN4 and its role in mediating human FSGS.

The α-actinins are 100 kD rod shaped proteins that form head-to-tail homodimers [18]. α-Actinin monomers contain three distinct domains: an N-terminal actin binding domain (ABD), four spectrin-like repeats (SRs), and a C-terminal EF hands (calmodulin like domain). There are four human α-actinin (ACTN1-4) family members, encoding closely related actin crosslinking proteins. ACTN2 and ACTN3 (calcium insensitive) show sarcomere-limited expression [19]. The non-muscle cytoskeletal ACTN1 (highly calcium sensitive) and ACTN4 (moderately calcium sensitive), appear to be widely expressed [20, 21]. However, for unclear reasons, the human phenotype associated with ACTN4 mutations is apparent only in the kidney [12]. Additionally, ACTN1 mutations were identified as a cause of congenital macrothrombocytopenia, an inherited disorder showing low platelet counts [22]. ACTN2 mutations have been found in rare families with hypertrophic cardiomyopathy [23]. The ACTN3 R577X variant, leading to absence of ACTN3 protein due to an early stop-codon, is absent in 18% of healthy white individuals [24]. There is no apparent disease phenotype as a result of this variant. However, the homozygous genotype appears with very low frequency in elite sprint athlete [25]. The loss of ACTN3 is associated with reduced skeleton high velocity contraction. Altogether, these studies revealed unique roles of different α-actinin isoforms different tissues.

In addition to bundling F-actin, ACTN4 interacts with various other proteins, consistent with multiple roles in cell function. Some of these studies were conducted either using crude extraction of α-actinin or anti-α-actinin antibodies that are not specific for ACTN4. For example:

1. *Cell adhesion* α-actinins interact directly with β1-integrin [26, 27], vinculin [28, 29], zyxin [30], kindlin-1 [31] to modulate focal adhesion, and to link the cytoskeleton to the extracellular matrix.
2. *Cell junction* The glomerular slit diaphragm between podocyte foot processes shares many morphologic features with the adherens junction seen in epithelial cells. The glomerular slit diaphragm composes of P-cadherin, α-catenin, β-catenin, γ-catenin, and ZO-1 [32]. α-actinins have been shown to interact directly with α-catenin [33]. Moreover, the slit-diaphragm protein nephrin appears to form a major structural component of the slit diaphragm between adjacent podocytes. ACTN4, together with five other cell junction proteins, including the membrane-associated guanylate kinase inverted 2_synaptic scaffolding molecule (MAGI-2/S-SCAM), IQ motif-containing GTPase activating protein1 (IQGAP1), αII spectrin, and βII spectrin was detected as part of the neprhin multiprotein complex [34]. Therefore, ACTN4 could serve as a linker between F-actin and other adherens junction proteins.
3. *Cell signaling* One of the key signaling molecules phosphatidylinositol 4,5-bisphosphate (PIP2) was shown to bind α-actinin at the plasma membrane. Upon PI3 Kinase (PI3K) activation, PIP2 is converted to phosphatidylinositol 3,4,5-trisphosphate (PIP3), which decreases the association of α-actinin to actin filament within stress fibers, as well as the association rate to integrin within focal adhesions [35, 36]. Moreover, ACTN4 can interact PI3K downstream kinase Akt to mediate cell proliferation [37]. Finally, PIP3 binding ACTNs also increases their susceptibility to calpain-1 and -2-mediated proteolysis [38].
4. *Nuclear transcription activator* ACTN4 was reported to be present in cell nuclei in 1998 [39]. Notably, PI3K inhibition and actin depolymerization both promote nuclear accumulation of ACTN4. Since then, ACTN4 has been reported to be a transcription activator of the estrogen receptor (ERα) [40, 41], the retinoic acid receptor (RAR) [42], the myocyte enhancer factor (MEF) [43, 44], the vitamin D receptor [41], the androgen receptor [45], NF-ķB transcription factors [46], thus potentially regulating transcription activity of multiple genes.

### ACTN4 mutations in humans with FSGS

Positional cloning approaches identified three different point mutations in ACTN4 as the cause of FSGS in three unrelated families [12]. These families show autosomal dominant inheritance of disease, with high but incomplete penetrance and variable expressivity. Most of the affected individuals initially presented with low-grade proteinuria mainly in early adulthood with declining kidney function that slowly progressed to ESRD. The K255E, T259I, and S262P mutations identified in these families are all located within the evolutionarily conserved ABD of ACTN4. As assessed by actin filament co-sedimentation assays, all mutations are associated with increased ACTN4 binding affinity to F-actin. ACTN4, but not ACTN1, is expressed at high levels (as detected by western blot) in human kidney. By immunofluorescence staining, it was found that ACTN4 is most prominently distributed in podocytes, with some distribution in other vasculature in the renal cortex. These findings are consistent with previous reports, suggesting that ACTN4 is highly expressed in podocytes, and with less expression elsewhere within the kidney [47, 48].

Two additional missense mutations in the ABD, W59R and I149del, were subsequently defined as disease-causing based on the following criteria: (1) mutants formed abnormal cellular aggregates with F-actin within the cell; (2) these mutations increased binding affinity of ACTN4 to F-actin, (3) ACTN4 with these mutations co-segregated with affected individuals within families; (4) the mutations occur in evolutionarily-conserved ABD domain [49]. ACTN4 mutations (five total disease-causing mutations including the three previously discovered) accounted for approximately 3.5% of 141 familial FSGS cases screened in this study. The W59R substitution identified in this study was of particular interest as it was a de novo mutation not present in the proband’s parent. This individual exhibited proteinuria at age 5 and progressed to ESRD within 3 years, much earlier than other families with ACTN4 mutations. In addition, this individual developed recurrent proteinuria and FSGS after transplantation, the only such case reported to date.

**Fig. 1:**
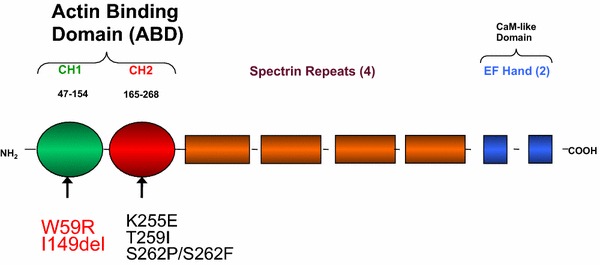
Functional domains of the human ACTN4 protein. The actin-binding domain (ABD) consists of CH1 (amino acid 47–154) and CH2 domain (amino acid 165–268). Mutations within ACTN4 including W59R and I149del in the CH1 domain and K255E, T259I, S262P and S262F in the CH2 domain have been associated with human FSGS.

Another clinical report documented a germline mosaicism ACTN4 mutation at S262F in the father of two affected siblings [50]. This mutation occurs at the same location as the previously reported heterozygous mutation S262P. Both patients developed FSGS in early childhood (3–4 years old) and rapidly progressed to ESRD. Renal biopsy showed a collapsing variant of FSGS in one affected sibling, a histological subtype of FSGS associated with a poorer prognosis. All of the disease causing ACTN4 mutations and their location in ABD has been summarized in Fig. 1 [51, 52].

### Actn4 mouse models

Kos et al. generated Actn4 knockout (Actn4 KO) mice [53]. A significant percentage of homozygous Actn4 KO mice suffer perinatal death. The remaining survivors go on to develop albuminuria and FSGS at about 10 weeks of age. Transmission electron microscopy of these Actn4 KO mice shows podocyte foot-process effacement. There is no clear alteration in podocin, nephrin, or type IV collagen expression in homozygous Actn4 KO mice by immunofluorescence staining. Even though strong expression of both Actn1 and Actn4 are detected in wild type (WT) mouse podocytes, the loss of Actn4 alone leads to a significant renal phenotype, suggesting that Actn4 plays a nonreduntant role in the mouse kidney. Additionally, homozygous Actn4 KO mice also show a decrease in the number of podocytes in the glomeruli, and a significant increased number of podocytes shedding in the urine as indicated by the presence of podocyte marker WT-1 protein [54]. Podocytes isolated from homozygous Actn4 KO mice showed decreased phosphorylation of β1-integrin and weaker integrin-cytoskeleton linkages. Consequently, They also showed decreased attachment in response to shear stress in culture. Altogether, homozygous Actn4 KO mice demonstrated the importance of the expression level of Actn4 in kidney disease. Indeed, Liu et al. reported that decreased expression of ACTN4 could occur in humans with primary glomerulopathies including sporadic FSGS, minimal change disease, and IgA nephropathy [55].

Yao et al. generated Actn4 K256E (a mutation analogous to the FSGS-causing K255E mutation in humans) knock-in (Actn4 KI) mice in an effort to mimic the human phenotype [56]. Homozygous Actn4 KI mice also showed perinatal lethality, albuminuria, and podocyte foot enfacement as seen in the Actn4 KO mice. Henderson et al. further examined the pathological characterization of both K256E and KO mice [57]. Both homozygous KI and KO mice exhibit the phenotype of collapsing glomerulopathy. Actn4 KI and KO mice did not survive past 21 and 15 weeks respectively. Homozygous KI mice and all KO mice show decreased expression of podocyte differentiation markers, including WT-1 and synaptopodin, and increased proliferation markers, including cyclin D1 and KI-67. Heterozygous Actn4 KI mice represent a more genetically faithful model for the autosomal-dominant ACTN4-mediated FSGS in humans. These mice did not develop glomerulosclerosis during the 70-week study period. However, they did exhibit focal glomerular hypertrophy and mild glomerular ultrastructural abnormalities, including mild podocyte cell body abnormalities, glomerular basement membrane (GBM) thickening and redundancy, and diffuse electron-dense aggregate accumulation. These abnormalities in heterozygous Actn4 KI mice may increase susceptibility to injury caused by other genetic or environmental stressors.

Michaud et al. generated podocyte-specific (driven by murine nephrin promoter) transgenic mice that overexpressed the K256E mutation (homologus to the human K255E mutation) [58]. Podocyte specific K256E mutant (K256E-Actn4^pod^) mice show heterogeneity, potentially due to variation in the expression of the transgene. The transgenic mice that had higher expression of mutant Actn4 exhibited significant albuminuria, glomerulosclerosis, and foot process enfacement at 10 weeks of age. These proteinuric transgenic mice also showed reduced expression of nephrin. Both proteinuric and non-proteinuric Actn4 transgenic mice exhibited increased average systolic BP measured by tail-cuff plethysmography. Michaud et al. also generated podocyte-specific transgenic mice overexpressing the wild-type Actn4 (WT-Actn4^pod^) as a control line of mice. They showed that WT-Actn4^pod^ mice are indistinguishable from their nontransgenic littermates [59]. These mice do not develop albuminuria, glomerulosclerosis, foot process enfacement, or elevated systemic blood pressure. The authors concluded that it is the K256E Actn4 mutation, not the overexpression of Actn4, underlies the FSGS phenotype in K256E-Actn4^pod^.

### Mechanisms by which ACTN4 mutations lead FSGS

Several studies have examined the effect of ACTN4 mutations on protein function in vitro. Weins et al. found that K255E mutant ACTN4 (full length or ABD) shows greater binding affinity to F-actin compared to WT ACTN4 based on co-sedimentation studies [60]. The addition of Ca^2+^ decreased the binding affinity of WT ACTN4 to F-actin, but not K255E ACTN4. These results suggest that K255E mutant ACTN4 binding to F-actin is not subject to Ca^2+^ regulation in vitro. One hypothesis proposed in this study is that a conformational change occurs when mutant K255E binds to F-actin, resulting in greater binding affinity. Interestingly, the crystal structure of the ABD of mutant K255E ACTN4 in absence of actin shows the same conformation as the WT protein [52]. Perhaps, the F-actin bound form of ACTN4 adopts a different structural conformation than ACTN4 alone. Galkin et al. used cryo-electron microscopy reconstruction (relative low resolution) of F-actin cross-linked by ABD of ACTN4 to build a model. They predicted that the bound form of ACTN4 would adopt a different conformation than the unbound ACTN4 crystal structure to enable binding to F-actin [61]. Therefore, the high-resolution cryo-electron microscopy structure of F-actin cross-linked by mutant ACTN4 is needed to better explain how mutations lead to binding affinity changes of ACTN4 to F-actin, and to visualize the exact conformational changes.

Weins et al. used electron microscopy to examine the appearance of actin filaments cross-linked either by WT or K255E mutant ACTN4 in vitro [60]. They showed that WT ACTN4 crosslinks actin filaments into thick parallel bundles with defined spacing. On the other hand, mutant ACTN4 induces the formation of a disordered and entangled network of thin filament bundles. Similarly, under fluorescent microscopy, actin filaments (fluorescently labeled) cross-linked by WT ACTN4 form an evenly spaced and finely reticulated actin network. In comparison, actin filaments cross-linked by K255E mutant ACTN4 form a more coarsely reticulated network with a smaller mesh size [62]. Based on bulk rheology experiments, Ward et al. suggested that the dissociation of mutant K255E ACTN4 from actin is much slower than the WT ACTN4. Additionally, Yao et al. observed that the actin network cross-linked by K255E mutant ACTN4 is more brittle, with a lower breaking stress in comparison to networks cross-linked with WT [63]. Together, these in vitro studies reflect how mutations within ACTN4 change the intrinsic biochemical and biophysical properties of the protein.

The effects of K255E mutant ACTN4 on cellular functions have also been examined. Using immortalized lung fibroblasts and podocytes isolated from homozygous Actn4 KI mice, Weins et al. observed that mutant Actn4 aggregates with F-actin [60]. Biopsies from patients with ACTN4 mutations also show segmental weak linear, irregular granular, and punctuate appearance of ACTN4 staining surrounding the glomerular capillary wall [64]. These abnormalities could potentially be direct results of increased binding affinity to F-actin of mutant actn4, resulting in disruption of actin reorganization or assembly. Additionally, Weins et al. also reported that two other known interacting proteins cortactin and synaptopodin were also present in the mutant Actn4/F-actin aggregates [60]. The sequestration of these binding partners, which play important roles in podocyte function, could alter the response of podocytes to environmental stressors. Michaud et al. used conditionally immortalized mouse podocytes infected with adenoviral constructs containing WT or K256E murine Actn4 to assess the functional consequence of mutations on subcellular localization, adhesion, spreading, migration, and formation of foot process-like peripheral projections [65]. They found that mutant K256E Actn4 was detected predominantly in the Triton-insoluble fraction of cellular extraction, and localizes almost exclusively along actin stress fibers. On the other hand, WT Actn4 was detected primarily in the Triton-soluble fraction of cellular extraction, localized to membrane-associated cortical actin and focal adhesions, with some expression along stress fibers. Podocytes overexpressing WT or mutant K256E Actn4 show similar adhesion to the extracellular matrix (collagen-I). However, podocytes overexpressing mutant K256E Actn4 exhibit significantly reduced ability to spread and migrate on collagen-I, and have a reduced mean number of actin-rich peripheral projections (reminiscent of podocyte foot processes in cell culture) compared to WT, perhaps all due to mutant Actn4/F-actin aggregates formation.

The sequestration of mutant ACTN4 together with F-actin in cellular aggregates can prevent ACTN4 to serve as transcriptional co-regulator. ACTN4 harbors a functional nuclear receptor interaction motif LXX LL (where L is leucine, X can be any amino acids) [41]. Khurana el al reported that a portion of WT ACTN4 can move to the podocyte nucleus and stimulate nuclear RARα [42] and NF-ķB [46] mediated transcription. When the LXXLL motif is mutated to LXXAA, WT ACTN4 (LXX AA) exhibits significant loss of the ability to potentiate RARα mediated transcription. On the other hand, disease-causing mutant ACTN4, predominantly cytoplasmic, failed to translocate to the nucleus and show an inability to stimulate RARα-mediated transcription.

Mutations within ABD of ACTN4 could lead to greater degradation of the protein in the cell [56]. Yao et al. reported that homozygous Actn4 KI mice showed markedly decreased expression of Actn4 protein in homozygous KI mice, and moderately decreased expression in heterozygous KI mice. Conditionally immortalized fibroblasts isolated from WT and homozygous K256E mice were used to compare actn4 protein synthesis and degradation rates in the cell. They found that the protein degradation rate of mutant Actn4 is much faster that WT Actn4. There is no difference in protein synthesis rates. The greater Actn4 degradation rate could potentially explain the lower level of Actn4 protein in homozygous KI mice compared to WT mice in kidney, lung, liver and brain. Rapid degradation of Actn4 in mutant fibroblasts can be reversed by the treating of a selective proteasome inhibitor lactacystin. This result suggests that mutant Actn4 may be degraded through the ubiquitin–proteasome pathway. Interestingly, it was reported that ubiquitin C-terminal hydrolase L1 (UCHL1) is up regulated in a subset of human glomerulopathies, including primary FSGS [66]. Reed et al. intercrossed K256E-Actn4^pod^ with heterozygous UCHI1 KO mice [67]. They found that mice that are heterozygous for an Actn4 transgene but homozygous for UCHL1 KO (K256E-Actn4^pod+^/UCHL1^−/−^) exhibited significantly ameliorated albuminuria, glomerulosclerosis, and foot process enfacement at 10 weeks of age. This result suggested that preserving K255E/WT Actn4 heterodimers from proteolysis might help to maintain podocyte function.

Recently, Grgic et al. used translating ribosome affinity purification (TRAP) to isolate and compare podocyte-specific mRNA expression between heterozygous Actn4 KI mice and WT [68]. They found that among other up-regulated genes in heterozygous Actn4 KI mice, myotonic dystrophy protein kinase (Dmpk), a Rho-associated serine-threonine protein kinase, is also upregulated at the protein level in human FSGS kidney biopsies. The role of DMPK in the development of FSGS in humans has yet to be elucidated.

## Conclusions

Mutations in the ACTN4 gene cause a highly penetrant, autosomal dominant form of familial FSGS in humans. Studies in Actn4 KO, Actn4 KI, and transgenic Actn4 mouse models confirm the importance of this gene in kidney function. Even though several studies have shown that ACTN4 mutations lead to biochemical, biophysical, and cellular functional changes, more research is needed to elucidate the exact pathways by which these mutations lead to podocyte injury, which may provide insights into therapeutic development. Since highly penetrant Mendelian forms of kidney disease caused by mutations in ACTN4 and other genes are rare examples where we can unequivocally identify the cause, the development of the specific therapies treating these diseases will be a proof of concept to advance individualized treatment in nephrology.

## Abbreviations

ACTN4: alpha-actinin-4 gene
FSGS: familial focal segmental glomerulosclerosis
ABD: actin-binding domain
NS: nephrotic syndrome
ESRD: end stage renal disease
INF2: inverted formin 2
TRPC6: canonical transient receptor potential 6
NPHS1: nephrin
NPHS2: podocin
SRs: spectrin-like repeats
MAGI-2/S-SCAM: membrane-associated guanylate kinase inverted 2_synaptic scaffolding molecule
IQGAP1: IQ motif-containing GTPase activatingprotein1
PIP2: phosphatidylinositol 4,5-bisphosphate
PI3K: PI3 kinase
PIP3: phosphatidylinositol 3,4,5-trisphosphate
Actn4 KO: actn4 knockout
WT: wild type
Actn4 KI: actn4 K256E knock-in
GBM: glomerular basement membrane
K256E-Actn4^pod^: podocyte specific K256E mutant
UCHL1: ubiquitin C-terminal hydrolase L1
TRAP: translating ribosome affinity purification
Dmpk: myotonic dystrophy protein kinase
